# PARPAL: PARalog Protein redistribution using Abundance and Localization in yeast database

**DOI:** 10.1093/g3journal/jkaf148

**Published:** 2025-06-28

**Authors:** Brittany M Greco, Gerardo Zapata, Rohan Dandage, Mikhail Papkov, Vanessa Pereira, François Lefebvre, Guillaume Bourque, Leopold Parts, Elena Kuzmin

**Affiliations:** Department of Biology, Concordia University, 7141 Sherbrooke St W, Montreal, QC, Canada H4B 1R6; Centre for Applied Synthetic Biology, Centre for Structural and Functional Genomics, Concordia University, 7141 Sherbrooke St W, Montreal, QC, Canada H4B 1R6; Canadian Centre for Computational Genomics (C3G), McGill University, 1010 Sherbrooke St W, Montreal, QC, Canada H3A 2R7; Victor Phillip Dahdaleh Institute of Genomic Medicine, McGill University, 740 Dr Penfield Ave, Montreal, QC, Canada H3A 0G1; Department of Biology, Concordia University, 7141 Sherbrooke St W, Montreal, QC, Canada H4B 1R6; Centre for Applied Synthetic Biology, Centre for Structural and Functional Genomics, Concordia University, 7141 Sherbrooke St W, Montreal, QC, Canada H4B 1R6; Institute of Computer Science, University of Tartu, Narva mnt 18, 51009 Tartu, Estonia; Department of Biology, Concordia University, 7141 Sherbrooke St W, Montreal, QC, Canada H4B 1R6; Centre for Applied Synthetic Biology, Centre for Structural and Functional Genomics, Concordia University, 7141 Sherbrooke St W, Montreal, QC, Canada H4B 1R6; Canadian Centre for Computational Genomics (C3G), McGill University, 1010 Sherbrooke St W, Montreal, QC, Canada H3A 2R7; Victor Phillip Dahdaleh Institute of Genomic Medicine, McGill University, 740 Dr Penfield Ave, Montreal, QC, Canada H3A 0G1; Canadian Centre for Computational Genomics (C3G), McGill University, 1010 Sherbrooke St W, Montreal, QC, Canada H3A 2R7; Victor Phillip Dahdaleh Institute of Genomic Medicine, McGill University, 740 Dr Penfield Ave, Montreal, QC, Canada H3A 0G1; Department of Human Genetics, McGill University, 3640 University, Montreal, QC, Canada H3A 0C7; Institute of Computer Science, University of Tartu, Narva mnt 18, 51009 Tartu, Estonia; Wellcome Sanger Institute, Wellcome Genome Campus, Hinxton, Cambridgeshire CB10 1SA, United Kingdom; Department of Biology, Concordia University, 7141 Sherbrooke St W, Montreal, QC, Canada H4B 1R6; Centre for Applied Synthetic Biology, Centre for Structural and Functional Genomics, Concordia University, 7141 Sherbrooke St W, Montreal, QC, Canada H4B 1R6; Department of Human Genetics, McGill University, 3640 University, Montreal, QC, Canada H3A 0C7; Rosalind and Morris Goodman Cancer Institute, McGill University, 1160 Pine Ave W, Montreal, QC, Canada H3A 1A3

**Keywords:** budding yeast, *Saccharomyces cerevisiae*, paralogs, duplicated genes, protein abundance, protein subcellular localization, phenomics, high-content screening, deep neural network

## Abstract

Whole-genome duplication (WGD) events are common across various organisms; however, the retention and evolution of WGD paralogs is not fully understood. Quantitative measure of protein redistribution in response to the deletion of their WGD paralog provides insight into sources of gene retention. Here, we describe PARPAL (PARalog Protein Redistribution using Abundance and Localization in Yeast), a web database that houses results of high-content screening and deep learning neural network analysis of the redistribution of 164 proteins reflecting how their subcellular localization and protein abundance change in response to their paralog deletion in the budding yeast, *Saccharomyces cerevisiae*. We interrogated a total of 82 paralog pairs in 2 genetic backgrounds for a total of ∼3,500 micrographs of ∼460,000 cells. For example, Skn7–Hms2 exhibited dependent redistribution, and Cue1–Cue4 showed compensatory redistribution response. PARPAL also links to other studies on trigenic interactions, protein–protein interactions and protein abundance. PARPAL is available at https://parpal.c3g-app.sd4h.ca and is a valuable resource for the yeast community interested in understanding the retention and evolution of paralogs and can help researchers to investigate protein dynamics of paralogs in other organisms.

## Introduction

Gene duplication events are prevalent across the tree of life, from single-cell organism, such as bacteria, to eukaryotic multicellular complex organisms, such as humans (Kuzmin *et al*. 2022). These events result from polyploid events resulting in whole-genome duplicates (WGDs) or small-scale duplicates (SSDs; Kuzmin *et al*. 2022). Although most duplicated genes are removed from the population, there is a significant number of paralogs that are retained through several mechanisms: neofunctionalization, whereby 1 paralog accumulates mutations and acquires a new function; subfunctionalization, where paralogs partition their functional domains; and dosage amplification or back-up compensation (Kuzmin *et al*. 2022).

The budding yeast, *Saccharomyces cerevisiae* underwent 1 round of WGD where 551 paralogs have been retained (Byrne and Wolfe 2005). Duplicated genes are thought to provide genetic robustness; and thus, they are an important subset of genes to study (Gu *et al*. 2003). Previous genetic interaction studies have shown that yeast exhibits a greater fitness defect and many trigenic interactions when both paralogs are deleted, revealing a compensatory relationship between paralogs (VanderSluis *et al*. 2010; Kuzmin *et al*. 2020). On the other hand, a dependency relationship between paralogs was observed whereby a protein's function is dependent on its paralog, which often occurs in heteromeric paralogs that interact physically (Diss *et al*. 2017).

In a previous study, we developed a high-content microscopy approach with single-cell resolution to understand the compensatory and dependency dynamics of paralogous proteins (Dandage *et al*. 2025). Three hundred and twenty-eight strains harboring a GFP-tagged protein in the wild-type or deletion background of its paralog, for a total of 164 unique GFP proteins comprising 82 paralog pairs, were imaged and computationally analyzed to detect a change in protein dynamics. Three different analysis methods were taken to assess protein redistribution, capturing changes in subcellular localization and abundance in wild-type or deletion genetic backgrounds of their paralogs: visual inspection, protein abundance quantification, and redistribution analysis. Deep neural network was used to extract 128 features and quantify redistribution. Several aspects are captured in Dandage *et al*. study: how often do proteins change their localization upon the deletion of their paralog, how often is this localization compensatory or dependent, which proteins are more likely to exhibit this localization, what drives relocalization, and which features are predictive of protein redistribution. In total, they found that 32 proteins exhibited a high redistribution score, of which 30 proteins showed a change in protein abundance and 9 changed subcellular localization indicating a compensatory or dependent response (Dandage *et al*. 2025). In addition, 1 protein that exhibited a subcellular localization change by visual inspection and 14 proteins that showed a protein abundance change did not receive a high redistribution score. The nonoverlapping proteins were associated with a lower magnitude of protein abundance change and represented false negatives of the deep neural network scoring approach, which was more powered to identify subcellular localization changes. Compensation was observed when a protein increased in abundance and/or changed its subcellular localization to be more similar to its paralogous protein upon the deletion of that paralog. Dependency was observed when a protein decreased in abundance and/or changed its subcellular localization to be different from its own or that of its paralogous protein, which is deleted.

To enable easy access to our microscopy images of the subcellular localization and abundance changes of proteins in response to their paralog perturbation, we developed a web-accessible database called PARPAL (PARalog Protein Retribution using Abundance and Localization in Yeast). PARPAL currently contains ∼3,500 micrographs of ∼460,000 cells. PARPAL also links with *S. cerevisiae* Database (SGD; Wong *et al*. 2023) and integrates Kuzmin *et al*. (2020; trigenic interaction study), Diss *et al*. (2017; protein–protein interaction [PPI] study) and DeLuna *et al*. (2010; protein abundance study) allowing for integration of multiple datasets related to yeast paralogs. This new database can be used to explore the relationship between paralog gene products in the budding yeast and differs from other databases as this catalog of information can also be used as a resource to refer to and compare findings from other studies as well as provide insight into protein dynamics of paralogs in other eukaryotic organisms.

## Materials and methods

### Phenomic screen and analysis

Phenomic screens and analyses were conducted as described elsewhere (Dandage *et al*. 2025).

### Database system construction

PARPAL was developed to facilitate the navigation of single-cell paralog pair images and their corresponding protein abundance and subcellular localization changes. PARPAL is a web application using Shiny's (Chang *et al*. 2024) framework built with python (mainly using common packages such as scikit-image, pandas, numpy, and matplotlib). The app plots tables and renders images hosted by the Canadian Centre for Computational Genomics within the S4DH's (Secure Data for Health) secure cloud infrastructure.

## Results and discussion

### Microscopy data acquisition

**Fig. 1.**
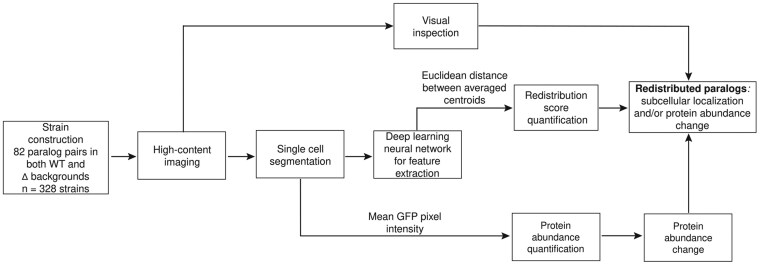
Overview of the workflow for identifying redistributed paralogous proteins using subcellular localization and protein abundance change.

Paralog pairs available on the PARPAL database were chosen from genes originating from WGD (Byrne and Wolfe 2005) and showed differences in their subcellular localization (Huh *et al*. 2003; Chong *et al*. 2015; Koh *et al*. 2015) resulting in 82 paralog pairs. The list includes 81 WGD paralog pairs and 1 (*HTA1-HTA2*) originating from an older duplication event (Tables 1 and 2). In total, 328 yeast query strains were constructed harboring 164 GFP fusion proteins in 2 genetic backgrounds: wild type and deletion of their paralog. Micrographs were acquired using an automated spinning disk confocal microscope with a 60× water-immersion objective (Evotec Opera, PerkinElmer). Four images per strain per replicate containing 50 to 100 cells were captured in a single plane, totaling ∼3,500 images. These images then underwent analysis using a deep learning neural network approach for single-cell segmentation, capturing ∼460,000 segmented single cells. Image analysis steps consisted of image annotation and processing, neural network architecture and training, post processing for segmentation, and filtering of image data. The annotation process effectively separated individual cells from cell clumps and other imaging artifacts. Image processing allowed for the calculation of a reference background image and subtraction from all images to standardize the pixel intensity within all the images and reduce noise. A neural network was trained and used to segment cells within micrographs. A postprocessing algorithm was additionally used for individual cells that did not perfectly segment previously. Finally, objects <256 and >8,192 pixels by area were removed. Together these steps ensured that proper single-cell segmentation was achieved. Images were filtered out based on a mismatch of localization between our study and a previous study (Chong *et al*. 2015). Images with abnormalities, artifacts, and high heterogeneity, as well as images with very low cell numbers, were also excluded. A second deep learning neural network was used for the redistribution analysis, by extracting 128 features to capture protein abundance and subcellular localization change. Protein abundance change was also scored separately using mean GFP pixel intensities from the resulting segmented images. The workflow is shown in Fig. 1.

**Table 1.** Summary statistics for all screens stored in PARPAL.

Genetic background	Paralog 1	Paralog 2	Replicates	No. of proteins	No. of micrographs	No. of cells
Wild type	Paralog 1-GFP	Paralog 2 wild type	3	82	892	104,899
Wild type	Paralog 1 wild type	Paralog 2-GFP	3	82	853	96,230
Deletion	Paralog 1-GFP	Paralog 2 deletion	3	82	888	102,587
Deletion	Paralog 1 deletion	Paralog 2-GFP	3	82	851	157,235
			Total	328	3,484	460,951

**Table 2.** The redistribution score, protein abundance, and localization change for all paralog pairs.

Paralog pair	ORF1-ORF2	Gene-GFP	ORF-GFP	Redistribution score^a^	Redistribution result^a^	Relative abundance change, log_2_-fold change^b^	Relative abundance change, *q*-value^b^	Relative abundance change type^b^	Relocalization type^c^	Relocalization description^c^
AAP1-APE2	YHR047C-YKL157W	AAP1	YHR047C	0.53	False	0.04	0.009	ns	Unclassified	Unclassified
AAP1-APE2	YHR047C-YKL157W	APE2	YKL157W	3.02	False	0.09	0.000	ns	Unclassified	Unclassified
ACE2-SWI5	YLR131C-YDR146C	ACE2	YLR131C	2.16	False	−0.11	0.068	ns	Unclassified	Unclassified
ACE2-SWI5	YLR131C-YDR146C	SWI5	YDR146C	2.18	False	0.15	0.019	ns	Unclassified	Unclassified
AGP1-GNP1	YCL025C-YDR508C	AGP1	YCL025C	0.95	False	−0.15	0.461	ns	Unclassified	Unclassified
AGP1-GNP1	YCL025C-YDR508C	GNP1	YDR508C	4.64	False	0.11	0.252	ns	Unclassified	Unclassified
AIM20-SKG1	YIL158W-YKR100C	AIM20	YIL158W	1.04	False	−0.06	0.000	ns	Unclassified	Unclassified
AIM20-SKG1	YIL158W-YKR100C	SKG1	YKR100C	4.05	False	0.13	0.000	ns	Unclassified	Unclassified
APT1-APT2	YML022W-YDR441C	APT1	YML022W	5.12	True	−0.59	0.000	Dependency	Unclassified	Unclassified
APT1-APT2	YML022W-YDR441C	APT2	YDR441C	4.73	True	−0.37	0.000	Dependency	Unclassified	Unclassified
AST1-AST2	YBL069W-YER101C	AST1	YBL069W	2.17	False	−0.08	0.050	ns	Unclassified	Unclassified
AST1-AST2	YBL069W-YER101C	AST2	YER101C	2.75	False	0.06	0.000	ns	Unclassified	Unclassified
CIT1-CIT2	YNR001C-YCR005C	CIT1	YNR001C	0.81	False	−0.04	0.013	ns	Unclassified	Unclassified
CIT1-CIT2	YNR001C-YCR005C	CIT2	YCR005C	7.17	True	−0.17	0.000	ns	Unclassified	Unclassified
CLB3-CLB4	YDL155W-YLR210W	CLB3	YDL155W	2.51	False	0.21	0.000	Compensation	Unclassified	Unclassified
CLB3-CLB4	YDL155W-YLR210W	CLB4	YLR210W	3.26	False	0.17	0.000	ns	Unclassified	Unclassified
CPR2-CPR5	YHR057C-YDR304C	CPR2	YHR057C	1.77	False	−0.09	0.000	ns	Unclassified	Unclassified
CPR2-CPR5	YHR057C-YDR304C	CPR5	YDR304C	3.69	False	−0.07	0.009	ns	Unclassified	Unclassified
CPT1-EPT1	YNL130C-YHR123W	CPT1	YNL130C	5.59	True	−0.30	0.000	Dependency	Unclassified	Unclassified
CPT1-EPT1	YNL130C-YHR123W	EPT1	YHR123W	6.12	True	−0.10	0.000	ns	Unclassified	Unclassified
CRP1-MDG1	YHR146W-YNL173C	CRP1	YHR146W	2.06	False	−0.10	0.001	ns	Unclassified	Unclassified
CRP1-MDG1	YHR146W-YNL173C	MDG1	YNL173C	3.37	False	0.23	0.000	Compensation	Unclassified	Unclassified
CUE1-CUE4	YMR264W-YML101C	CUE1	YMR264W	1.93	False	0.16	0.000	ns	Unclassified	Unclassified
CUE1-CUE4	YMR264W-YML101C	CUE4	YML101C	9.35	True	1.60	0.000	Compensation	Compensation	ER to cytoplasm
CUP2-HAA1	YGL166W-YPR008W	CUP2	YGL166W	2.01	False	0.00	0.092	ns	Unclassified	Unclassified
CUP2-HAA1	YGL166W-YPR008W	HAA1	YPR008W	6.30	True	−0.04	0.000	ns	Unclassified	Unclassified
CYC1-CYC7	YJR048W-YEL039C	CYC1	YJR048W	3.76	False	−0.16	0.000	ns	Compensation	Mitochondria to cytoplasm
CYC1-CYC7	YJR048W-YEL039C	CYC7	YEL039C	3.12	False	0.01	0.000	ns	Unclassified	Unclassified
DCS1-DCS2	YLR270W-YOR173W	DCS1	YLR270W	2.74	False	−0.13	0.000	ns	Unclassified	Unclassified
DCS1-DCS2	YLR270W-YOR173W	DCS2	YOR173W	1.26	False	−0.02	0.285	ns	Unclassified	Unclassified
DLS1-DPB3	YJL065C-YBR278W	DLS1	YJL065C	3.27	False	0.08	0.000	ns	Unclassified	Unclassified
DLS1-DPB3	YJL065C-YBR278W	DPB3	YBR278W	2.37	False	0.18	0.000	ns	Unclassified	Unclassified
DNF1-DNF2	YER166W-YDR093W	DNF1	YER166W	3.95	False	−0.27	0.000	Dependency	Unclassified	Unclassified
DNF1-DNF2	YER166W-YDR093W	DNF2	YDR093W	4.25	False	−0.17	0.000	ns	Unclassified	Unclassified
EDC1-EDC2	YGL222C-YER035W	EDC1	YGL222C	1.41	False	0.07	0.112	ns	Unclassified	Unclassified
EDC1-EDC2	YGL222C-YER035W	EDC2	YER035W	4.71	False	−0.17	0.000	ns	Unclassified	Unclassified
EMI2-GLK1	YDR516C-YCL040W	EMI2	YDR516C	3.34	False	−0.03	0.117	ns	Unclassified	Unclassified
EMI2-GLK1	YDR516C-YCL040W	GLK1	YCL040W	2.63	False	0.02	0.063	ns	Unclassified	Unclassified
EMP46-EMP47	YLR080W-YFL048C	EMP46	YLR080W	6.37	True	0.23	0.000	Compensation	Unclassified	Unclassified
EMP46-EMP47	YLR080W-YFL048C	EMP47	YFL048C	1.25	False	0.02	0.436	ns	Unclassified	Unclassified
ESL1-ESL2	YIL151C-YKR096W	ESL1	YIL151C	2.58	False	0.08	0.000	ns	Unclassified	Unclassified
ESL1-ESL2	YIL151C-YKR096W	ESL2	YKR096W	3.95	False	0.09	0.000	ns	Unclassified	Unclassified
FAA1-FAA4	YOR317W-YMR246W	FAA1	YOR317W	6.24	True	1.17	0.000	Compensation	Unclassified	Unclassified
FAA1-FAA4	YOR317W-YMR246W	FAA4	YMR246W	3.16	False	−0.34	0.000	Dependency	Unclassified	Unclassified
FAT3-INA1	YKL187C-YLR413W	FAT3	YKL187C	2.32	False	0.12	0.000	ns	Unclassified	Unclassified
FAT3-INA1	YKL187C-YLR413W	INA1	YLR413W	3.63	False	−0.18	0.000	ns	Unclassified	Unclassified
FLC1-FLC3	YPL221W-YGL139W	FLC1	YPL221W	1.97	False	0.04	0.019	ns	Unclassified	Unclassified
FLC1-FLC3	YPL221W-YGL139W	FLC3	YGL139W	2.00	False	0.15	0.000	ns	Unclassified	Unclassified
FMP45-YNL194C	YDL222C-YNL194C	FMP45	YDL222C	2.62	False	0.14	0.000	ns	Unclassified	Unclassified
FMP45-YNL194C	YDL222C-YNL194C	YNL194C	YNL194C	1.93	False	0.03	0.087	ns	Unclassified	Unclassified
FRK1-KIN4	YPL141C-YOR233W	FRK1	YPL141C	1.54	False	−0.12	0.000	ns	Unclassified	Unclassified
FRK1-KIN4	YPL141C-YOR233W	KIN4	YOR233W	2.97	False	0.03	0.092	ns	Unclassified	Unclassified
GGA1-GGA2	YDR358W-YHR108W	GGA1	YDR358W	8.57	True	0.15	0.000	ns	Compensation	Cytoplasm to Golgi
GGA1-GGA2	YDR358W-YHR108W	GGA2	YHR108W	7.22	True	−0.50	0.000	Dependency	Unclassified	Unclassified
GIC1-GIC2	YHR061C-YDR309C	GIC1	YHR061C	5.74	True	−0.06	0.000	ns	Unclassified	Unclassified
GIC1-GIC2	YHR061C-YDR309C	GIC2	YDR309C	6.36	True	−0.30	0.000	Dependency	Unclassified	Unclassified
GIN4-KCC4	YDR507C-YCL024W	GIN4	YDR507C	1.09	False	−0.02	0.090	ns	Unclassified	Unclassified
GIN4-KCC4	YDR507C-YCL024W	KCC4	YCL024W	2.14	False	0.14	0.000	ns	Unclassified	Unclassified
GIP3-HER1	YPL137C-YOR227W	GIP3	YPL137C	3.40	False	0.00	0.000	ns	Unclassified	Unclassified
GIP3-HER1	YPL137C-YOR227W	HER1	YOR227W	3.47	False	0.17	0.000	ns	Unclassified	Unclassified
GLO2-GLO4	YDR272W-YOR040W	GLO2	YDR272W	1.84	False	0.16	0.000	ns	Unclassified	Unclassified
GLO2-GLO4	YDR272W-YOR040W	GLO4	YOR040W	2.37	False	0.07	0.000	ns	Unclassified	Unclassified
GPB1-GPB2	YOR371C-YAL056W	GPB1	YOR371C	4.50	False	−0.06	0.007	ns	Unclassified	Unclassified
GPB1-GPB2	YOR371C-YAL056W	GPB2	YAL056W	2.11	False	0.03	0.186	ns	Unclassified	Unclassified
GPM2-GPM3	YDL021W-YOL056W	GPM2	YDL021W	0.90	False	0.10	0.000	ns	Unclassified	Unclassified
GPM2-GPM3	YDL021W-YOL056W	GPM3	YOL056W	1.44	False	−0.07	0.974	ns	Unclassified	Unclassified
GRX1-GRX2	YCL035C-YDR513W	GRX1	YCL035C	2.29	False	0.23	0.000	Compensation	Unclassified	Unclassified
GRX1-GRX2	YCL035C-YDR513W	GRX2	YDR513W	2.56	False	0.04	0.000	ns	Unclassified	Unclassified
GSY1-GSY2	YFR015C-YLR258W	GSY1	YFR015C	4.04	False	0.27	0.000	Compensation	Unclassified	Unclassified
GSY1-GSY2	YFR015C-YLR258W	GSY2	YLR258W	5.83	True	0.34	0.000	Compensation	Unclassified	Unclassified
HBS1-SKI7	YKR084C-YOR076C	HBS1	YKR084C	5.62	True	−0.23	0.000	Dependency	Unclassified	Unclassified
HBS1-SKI7	YKR084C-YOR076C	SKI7	YOR076C	2.63	False	−0.13	0.000	ns	Unclassified	Unclassified
HMS2-SKN7	YJR147W-YHR206W	HMS2	YJR147W	8.39	True	0.19	0.000	ns	Dependency	Nucleus to cytoplasm
HMS2-SKN7	YJR147W-YHR206W	SKN7	YHR206W	1.69	False	0.05	0.000	ns	Unclassified	Unclassified
HTA1-HTA2	YDR225W-YBL003C	HTA1	YDR225W	6.41	True	0.19	0.000	ns	Unclassified	Unclassified
HTA1-HTA2	YDR225W-YBL003C	HTA2	YBL003C	3.49	False	−0.62	0.000	Dependency	Unclassified	Unclassified
ICY1-ICY2	YMR195W-YPL250C	ICY1	YMR195W	3.43	False	0.18	0.000	ns	Unclassified	Unclassified
ICY1-ICY2	YMR195W-YPL250C	ICY2	YPL250C	1.46	False	0.16	0.000	ns	Unclassified	Unclassified
INP52-INP53	YNL106C-YOR109W	INP52	YNL106C	2.63	False	0.15	0.000	ns	Unclassified	Unclassified
INP52-INP53	YNL106C-YOR109W	INP53	YOR109W	4.13	False	0.00	0.000	ns	Unclassified	Unclassified
JSN1-PUF2	YJR091C-YPR042C	JSN1	YJR091C	7.97	True	0.00	0.636	ns	Unclassified	Unclassified
JSN1-PUF2	YJR091C-YPR042C	PUF2	YPR042C	2.92	False	0.06	0.000	ns	Unclassified	Unclassified
LCB4-LCB5	YOR171C-YLR260W	LCB4	YOR171C	5.20	True	−0.10	0.000	ns	Unclassified	Unclassified
LCB4-LCB5	YOR171C-YLR260W	LCB5	YLR260W	3.47	False	0.28	0.000	Compensation	Unclassified	Unclassified
MHT1-SAM4	YLL062C-YPL273W	MHT1	YLL062C	1.64	False	0.01	0.419	ns	Unclassified	Unclassified
MHT1-SAM4	YLL062C-YPL273W	SAM4	YPL273W	4.12	False	0.55	0.000	Compensation	Unclassified	Unclassified
MIG2-MIG3	YGL209W-YER028C	MIG2	YGL209W	1.81	False	−0.11	0.000	ns	Unclassified	Unclassified
MIG2-MIG3	YGL209W-YER028C	MIG3	YER028C	3.86	False	−0.16	0.000	ns	Unclassified	Unclassified
MKK1-MKK2	YOR231W-YPL140C	MKK1	YOR231W	1.67	False	0.12	0.000	ns	Unclassified	Unclassified
MKK1-MKK2	YOR231W-YPL140C	MKK2	YPL140C	5.38	True	0.11	0.000	ns	Unclassified	Unclassified
MRX16-YLR108C	YDR132C-YLR108C	MRX16	YDR132C	2.15	False	0.04	0.002	ns	Unclassified	Unclassified
MRX16-YLR108C	YDR132C-YLR108C	YLR108C	YLR108C	2.20	False	0.10	0.000	ns	Unclassified	Unclassified
MSG5-SDP1	YNL053W-YIL113W	MSG5	YNL053W	2.69	False	0.06	0.000	ns	Unclassified	Unclassified
MSG5-SDP1	YNL053W-YIL113W	SDP1	YIL113W	1.35	False	0.03	0.003	ns	Unclassified	Unclassified
NSG1-NSG2	YHR133C-YNL156C	NSG1	YHR133C	1.32	False	0.07	0.001	ns	Unclassified	Unclassified
NSG1-NSG2	YHR133C-YNL156C	NSG2	YNL156C	3.06	False	0.17	0.000	ns	Unclassified	Unclassified
NTG1-NTG2	YAL015C-YOL043C	NTG1	YAL015C	2.24	False	−0.03	0.001	ns	Unclassified	Unclassified
NTG1-NTG2	YAL015C-YOL043C	NTG2	YOL043C	1.50	False	0.03	0.000	ns	Unclassified	Unclassified
NTH1-NTH2	YDR001C-YBR001C	NTH1	YDR001C	2.10	False	−0.05	0.675	ns	Unclassified	Unclassified
NTH1-NTH2	YDR001C-YBR001C	NTH2	YBR001C	2.77	False	−0.11	0.000	ns	Unclassified	Unclassified
OSM1-FRD1	YJR051W-YEL047C	FRD1	YEL047C	2.02	False	0.19	0.000	ns	Unclassified	Unclassified
OSM1-FRD1	YJR051W-YEL047C	OSM1	YJR051W	3.57	False	0.07	0.000	ns	Unclassified	Unclassified
PAM1-SVL3	YDR251W-YPL032C	PAM1	YDR251W	1.67	False	0.11	0.000	ns	Unclassified	Unclassified
PAM1-SVL3	YDR251W-YPL032C	SVL3	YPL032C	2.29	False	0.00	0.000	ns	Unclassified	Unclassified
PAP2-TRF5	YOL115W-YNL299W	PAP2	YOL115W	3.99	False	−0.03	0.045	ns	Unclassified	Unclassified
PAP2-TRF5	YOL115W-YNL299W	TRF5	YNL299W	6.63	True	0.07	0.421	ns	Unclassified	Unclassified
PCL10-PCL8	YGL134W-YPL219W	PCL10	YGL134W	2.16	False	0.14	0.000	ns	Unclassified	Unclassified
PCL10-PCL8	YGL134W-YPL219W	PCL8	YPL219W	2.33	False	0.14	0.000	ns	Unclassified	Unclassified
PCL6-PCL7	YER059W-YIL050W	PCL6	YER059W	1.29	False	0.00	0.735	ns	Unclassified	Unclassified
PCL6-PCL7	YER059W-YIL050W	PCL7	YIL050W	3.53	False	−0.14	0.000	ns	Unclassified	Unclassified
PDR1-PDR3	YGL013C-YBL005W	PDR1	YGL013C	1.85	False	0.09	0.496	ns	Unclassified	Unclassified
PDR1-PDR3	YGL013C-YBL005W	PDR3	YBL005W	2.53	False	0.06	0.000	ns	Unclassified	Unclassified
PEX30-PEX31	YLR324W-YGR004W	PEX30	YLR324W	5.07	True	−0.27	0.000	Dependency	Unclassified	Unclassified
PEX30-PEX31	YLR324W-YGR004W	PEX31	YGR004W	2.82	False	0.05	0.000	ns	Unclassified	Unclassified
PGM1-PGM2	YKL127W-YMR105C	PGM1	YKL127W	3.55	False	−0.17	0.000	ns	Unclassified	Unclassified
PGM1-PGM2	YKL127W-YMR105C	PGM2	YMR105C	3.11	False	0.39	0.000	Compensation	Unclassified	Unclassified
PHO87-PHO90	YCR037C-YJL198W	PHO87	YCR037C	2.68	False	0.05	0.000	ns	Unclassified	Unclassified
PHO87-PHO90	YCR037C-YJL198W	PHO90	YJL198W	3.17	False	−0.03	0.000	ns	Unclassified	Unclassified
POR1-POR2	YNL055C-YIL114C	POR1	YNL055C	2.86	False	−0.17	0.000	ns	Unclassified	Unclassified
POR1-POR2	YNL055C-YIL114C	POR2	YIL114C	12.72	True	0.74	0.000	Compensation	Dependency	Mitochondria to cytoplasm
RCR1-RCR2	YBR005W-YDR003W	RCR1	YBR005W	1.52	False	0.08	0.000	ns	Unclassified	Unclassified
RCR1-RCR2	YBR005W-YDR003W	RCR2	YDR003W	2.13	False	0.03	0.000	ns	Unclassified	Unclassified
RGA1-RGA2	YOR127W-YDR379W	RGA1	YOR127W	2.05	False	−0.21	0.000	Dependency	Unclassified	Unclassified
RGA1-RGA2	YOR127W-YDR379W	RGA2	YDR379W	3.09	False	0.14	0.000	ns	Unclassified	Unclassified
RGI1-RGI2	YER067W-YIL057C	RGI1	YER067W	1.79	False	0.06	0.082	ns	Unclassified	Unclassified
RGI1-RGI2	YER067W-YIL057C	RGI2	YIL057C	1.39	False	−0.19	0.000	ns	Unclassified	Unclassified
RPL40A-RPL40B	YIL148W-YKR094C	RPL40A	YIL148W	4.56	False	−0.24	0.000	Dependency	Unclassified	Unclassified
RPL40A-RPL40B	YIL148W-YKR094C	RPL40B	YKR094C	6.62	True	0.96	0.000	Compensation	Compensation	Cytoplasm to nuclear
RPS22A-RPS22B	YJL190C-YLR367W	RPS22A	YJL190C	7.68	True	−2.05	0.000	Dependency	Dependency	Nucleolar to cytoplasm
RPS22A-RPS22B	YJL190C-YLR367W	RPS22B	YLR367W	5.67	True	−0.84	0.000	Dependency	Dependency	Nucleolar to cytoplasm
RTN1-RTN2	YDR233C-YDL204W	RTN1	YDR233C	1.48	False	0.03	0.269	ns	Unclassified	Unclassified
RTN1-RTN2	YDR233C-YDL204W	RTN2	YDL204W	2.10	False	0.10	0.000	ns	Unclassified	Unclassified
SDS23-SDS24	YGL056C-YBR214W	SDS23	YGL056C	3.85	False	0.33	0.000	Compensation	Unclassified	Unclassified
SDS23-SDS24	YGL056C-YBR214W	SDS24	YBR214W	6.67	True	−0.27	0.000	Dependency	Unclassified	Unclassified
SEG2-SEG1	YKL105C-YMR086W	SEG1	YMR086W	4.72	False	0.10	0.000	ns	Unclassified	Unclassified
SEG2-SEG1	YKL105C-YMR086W	SEG2	YKL105C	3.64	False	0.01	0.032	ns	Unclassified	Unclassified
SIP3-LAM1	YNL257C-YHR155W	LAM1	YHR155W	6.34	True	−0.08	0.000	ns	Unclassified	Unclassified
SIP3-LAM1	YNL257C-YHR155W	SIP3	YNL257C	4.87	True	−0.04	0.461	ns	Unclassified	Unclassified
SOL1-SOL2	YNR034W-YCR073W-A	SOL1	YNR034W	3.22	False	0.23	0.000	Compensation	Unclassified	Unclassified
SOL1-SOL2	YNR034W-YCR073W-A	SOL2	YCR073W-A	5.13	True	0.15	0.000	ns	Unclassified	Unclassified
SRL1-SVS1	YOR247W-YPL163C	SRL1	YOR247W	3.12	False	−0.11	0.000	ns	Unclassified	Unclassified
SRL1-SVS1	YOR247W-YPL163C	SVS1	YPL163C	4.11	False	0.05	0.000	ns	Unclassified	Unclassified
STP3-STP4	YLR375W-YDL048C	STP3	YLR375W	1.98	False	0.04	0.003	ns	Unclassified	Unclassified
STP3-STP4	YLR375W-YDL048C	STP4	YDL048C	3.96	False	0.04	0.000	ns	Unclassified	Unclassified
UBX6-UBX7	YJL048C-YBR273C	UBX6	YJL048C	4.70	False	0.14	0.000	ns	Unclassified	Unclassified
UBX6-UBX7	YJL048C-YBR273C	UBX7	YBR273C	1.91	False	0.11	0.000	ns	Unclassified	Unclassified
UPA1-UPA2	YGR283C-YMR310C	UPA1	YGR283C	10.91	True	−0.14	0.000	ns	Dependency	Nuclear to cytoplasm
UPA1-UPA2	YGR283C-YMR310C	UPA2	YMR310C	6.30	True	−0.04	0.000	ns	Dependency	Nuclear to cytoplasm
URA10-URA5	YMR271C-YML106W	URA10	YMR271C	3.31	False	0.03	0.848	ns	Unclassified	Unclassified
URA10-URA5	YMR271C-YML106W	URA5	YML106W	2.55	False	0.07	0.000	ns	Unclassified	Unclassified
VHR1-VHR2	YIL056W-YER064C	VHR1	YIL056W	3.22	False	0.18	0.000	ns	Unclassified	Unclassified
VHR1-VHR2	YIL056W-YER064C	VHR2	YER064C	1.61	False	0.00	0.960	ns	Unclassified	Unclassified
VPS5-VPS501	YOR069W-YKR078W	VPS5	YOR069W	2.65	False	0.08	0.000	ns	Unclassified	Unclassified
VPS5-VPS501	YOR069W-YKR078W	VPS501	YKR078W	1.74	False	0.11	0.000	ns	Unclassified	Unclassified
YAP1801-YAP1802	YHR161C-YGR241C	YAP1801	YHR161C	4.44	False	0.20	0.000	ns	Unclassified	Unclassified
YAP1801-YAP1802	YHR161C-YGR241C	YAP1802	YGR241C	2.43	False	0.13	0.000	ns	Unclassified	Unclassified
YAP5-YAP7	YIR018W-YOL028C	YAP5	YIR018W	2.71	False	−0.10	0.501	ns	Unclassified	Unclassified
YAP5-YAP7	YIR018W-YOL028C	YAP7	YOL028C	1.13	False	−0.05	0.002	ns	Unclassified	Unclassified
YDR222W-YLR225C	YDR222W-YLR225C	YDR222W	YDR222W	1.61	False	−0.03	0.008	ns	Unclassified	Unclassified
YDR222W-YLR225C	YDR222W-YLR225C	YLR225C	YLR225C	1.02	False	0.00	0.796	ns	Unclassified	Unclassified
YEH1-YEH2	YLL012W-YLR020C	YEH1	YLL012W	1.66	False	0.12	0.000	ns	Unclassified	Unclassified
YEH1-YEH2	YLL012W-YLR020C	YEH2	YLR020C	3.08	False	0.03	0.000	ns	Unclassified	Unclassified
YHP1-YOX1	YDR451C-YML027W	YHP1	YDR451C	1.75	False	0.02	0.023	ns	Unclassified	Unclassified
YHP1-YOX1	YDR451C-YML027W	YOX1	YML027W	1.86	False	0.12	0.000	ns	Unclassified	Unclassified

The raw data summarized in this table are based on Dandage *et al*. (2025).

^a^Results derived from the analysis of deep neural network features.

^b^Results from mean GFP pixel intensity.

^c^Results derived from scoring by visual inspection. Redistribution result is “True” if the redistribution score is ≥4.73 and “False” if the redistribution score is <4.73. The relative protein abundance increase is significant when log_2_-fold change ≥ 0.2, *q* < 0.05, suggesting compensation, whereas the decrease is significant when log_2_-fold change ≤ −0.2, *q* < 0.05, suggesting dependency. Relocalization was classified by manual visual inspection. Compensation denotes proteins that respond to the loss of their paralog by changing their subcellular localization to reside in their paralog's compartment. Dependency denotes proteins that respond by diverging from their own or their paralogous protein's wild-type subcellular localization. Unclassified denotes proteins that are not detected by eye to change in their subcellular localization.

### Identification of redistributed paralogs

**Fig. 2.**
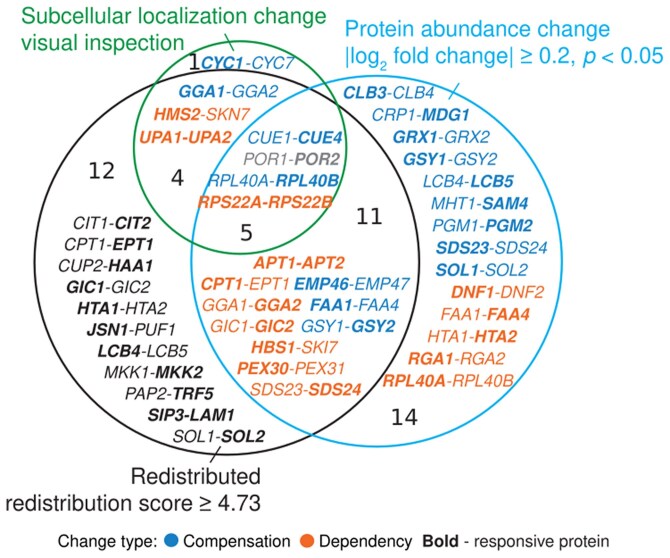
Venn diagram representing the overlap between scoring approaches. Proteins were classified as redistributed if the redistribution score is ≥4.73 by analyzing the deep neural network cell features (black circle). Proteins were classified exhibiting an abundance change when |log_2_-fold change| ≥ 0.2, *q* < 0.05 (light blue circle). Proteins were classified as exhibiting a subcellular localization change by visually inspecting their micrographs (green circle). Bolded genes encode responsive proteins. A compensatory response is denoted in blue, a dependent response is in orange, and mixed is in gray; black is unclassified.

Redistributed paralogs were identified using 3 different approaches: redistribution score quantification, visual inspection, and independent protein abundance quantification (Figs. 1 and 2, Table 2). An average using an arithmetic mean across each of 128 features for all cells in 3 replicates was used to generate 1 centroid point per protein in each genetic background. The redistribution score was then defined by the Euclidean distance between the centroid for a protein in a wild-type background and the centroid of the protein in a deletion background of its paralog. The threshold for redistribution scores was calculated to be 4.73 using an AUC–ROC analysis. A principal component analysis method was used to reduce dimensionality for visual representation of all 128 features. The mean GFP pixel intensity across all pixels in all the cell frames termed “abundance score” was used for protein abundance quantification in each genetic background. Abundance scores were first log-transformed, and the difference between the wild-type and deletion backgrounds was calculated to generate a relative abundance change. Manual visual inspection was also used to identify cases of subcellular localization change. A change in protein abundance was visually seen by a decrease or increase in brightness of GFP in the microscopy image. A change in localization was denoted by a visual inspection of a change in the pattern of the GFP signal within the cells of the image. This visual inspection provided an additional level of confidence to the machine learning approach to identify the patterns associated with protein localization and abundance change in response to the paralog deletion. Together these 3 approaches resulted in a set of redistributed paralogs capturing subcellular localization and/or protein abundance changes (Fig. 2). Since the redistribution score is based on the automated analysis of cell features, which are derived from the deep neural network, it is associated with a false-positive and false-negative rate. Thus, for example, there is a set of proteins that is scored as nonredistributed because its redistribution score falls below the threshold (4.73), that changes in either localization by manual visual inspection of the micrographs or protein abundance by mean GFP pixel intensity measure.

### Navigating through the PARPAL database

**Fig. 3.**
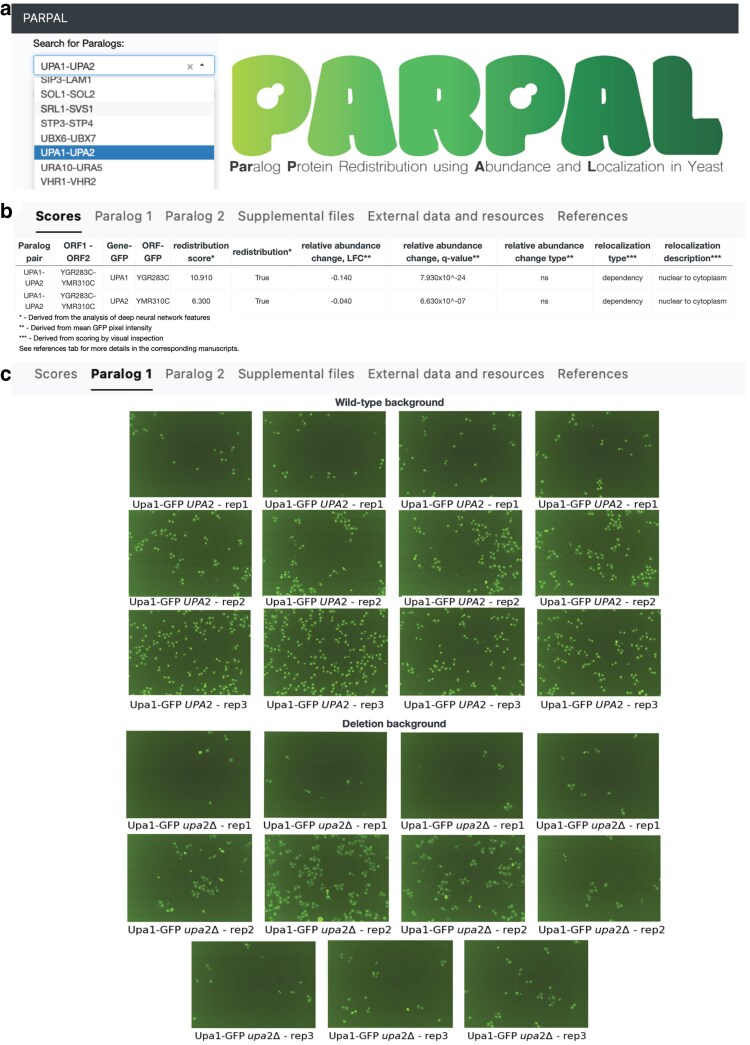
Screenshot of sample search and result page generated by PARPAL. a) A paralog pair of interest is chosen using the dropdown menu. b) Paralog pair, ORF1-ORF2, gene, ORF, redistribution score, redistribution, relative abundance change, LFC, relative abundance change, *q*-value, relative abundance change type, relocalization type (“ns” denotes no significant change), and relocalization description of each paralog protein (for example, Upa1 and Upa2) are displayed in the “Scores” tab. c) In the “Paralog 1” tab, micrographs of Upa1-GFP (Paralog1-GFP) are displayed in 2 genetic backgrounds: wild-type *UPA2 (PARALOG2)* and deletion-background *upa2*Δ (*paralog2*Δ). Similar to the “Paralog 1” tab, micrographs found in the “Paralog 2” tab are of Upa2-GFP (Paralog2-GFP) in both the wild-type *UPA1* (*PARALOG1)* and deletion *upa1*Δ (*paralog1*Δ) backgrounds (not shown).

The PARPAL web database stores redistribution scores as well as all the micrographs that were used to calculate this score. Using the pull-down menu, paralog pairs are chosen, and scores will automatically appear (Fig. 3a and b). Under the “Scores” tab, information such as “Paralog pair, ORF1-ORF2, gene, ORF, redistribution score, redistribution, relative abundance change LFC, relative abundance change *q*-value, relative abundance change type, relocalization type, and relocalization description” for each paralog is found (Fig. 3b). “Paralog pair” is the standard name of the paralog pair, *ORF1-ORF2* is the systematic name for the paralog pair and “Gene” and “ORF” represents the systematic and standard name associated with the GFP-tagged protein, respectively. Redistribution scores higher that 4.73 indicate proteins that redistribute in response to their paralog deletion, are annotated as “true” in the “redistribution” column. Redistribution scores below the 4.73 threshold indicate that proteins, which did not redistribute in response to their paralog deletion and are annotated as “false” in the “redistribution” column. The redistribution threshold was obtained based on the randomized controls and visual inspection. “Relative abundance change LFC” or “log_2_-fold change” refers to the change in protein abundance of the GFP-tagged protein that was calculated from the pixel intensity values of segmented images as previously described above by comparing the abundance in the genetic background of the wild-type and deletion allele of its paralog. The “relative abundance change *q*-value” is the FDR-corrected *P*-value and was calculated using the 2-sided Mann–Whitney *U* test and corrected for multiple testing using Benjamini–Hochberg method. “Relative abundance change type” shows whether the relative abundance change is significant if it passes the following threshold (|log_2_-fold change| ≥ 0.2, *P* < 0.05) and classified into “compensation” for paralogs that increase in protein abundance or “dependency” for paralogs that decrease in protein abundance when their paralog is deleted; “ns” indicates that no significant change in protein abundance at this threshold was detected. “Relocalization description” indicates a change in subcellular localization of the GFP protein in the absence of its paralog as evaluated by visual inspection. It reports the subcellular localization of the GFP protein in the genetic background of wild-type allele of its paralog and the subcellular localization of GFP protein in the genetic background of the deletion of its paralog in the following manner “subcellular localization A to subcellular localization B.” Relocalization description can also be “unclassified,” if the subcellular localization change was not detected by visual inspection. “Relocalization type” indicates the mechanism of relocalization so that if the mechanism is said to be “compensation,” the GFP protein changes subcellular localization to be more similar to the subcellular localization of its paralog when that paralog is deleted or “dependency,” if the GFP protein changes subcellular localization to be different than its own subcellular localization or of its paralog in response to the deletion of that paralog. If only 1 protein in the paralog pair exhibits redistribution, then only values for 1 protein will be reported, indicating that there is no redistribution for the paralog. In cases where no redistribution in either paralog pair is observed, the message “No redistribution or protein abundance change” will appear under the “Scores” tab.

Under the “Paralog 1” and “Paralog 2” tabs, micrographs for GFP proteins in the genetic background harboring a wild-type or deletion allele of its paralog can be found (Fig. 3c). The database allows zooming in and out, permitting a visual inspection of the microscopy images. Micrographs can be found in technical replicates captured from 4 fields of view as well as 3 biological replicates. All micrographs on PARPAL have the same microscopy settings. Some micrographs were filtered out during the quality control steps before analysis, as described above. The micrographs displayed for each GFP protein are displayed with the same threshold intensity settings to enable comparison. These parameters change across GFP proteins, and thus, they should not be used to compare GFP proteins to each other but rather to compare the same GFP protein between 2 different genetic backgrounds.

All supplemental information regarding all screen results housed in the PARPAL database is found in the tab called “Supplemental files.” The supplemental files consist of 5 tables and 2 datasets. Table S1 contains information regarding the yeast strains and plasmid used. Protein abundance for each paralog in each genetic background is found in Table S2. Mean protein abundance as well as protein abundance for each replicate for each paralog for each condition is also included in Table S2. Table S3 summarizes results of manual inspection. Table S4 includes all information for paralogs, including redistribution, relative protein abundance changes, and localization, which can also be found in the “Scores” tab for paralogs that report a true statement in the redistribution column. This table additionally includes this information for protein that did not exhibit redistribution upon the deletion of its paralog. Table S5 contains information on features that may be predictors of redistribution, such as PPIs, genetic interactions, and colocalization of their interactors. Data S1 and Data S2 contain the scores for all 128 features from the deep neural network and the protein abundance of every cell in every micrograph generated by this study, respectively.

### Exploring the protein dynamics of redistributed paralogs

**Fig. 4.**
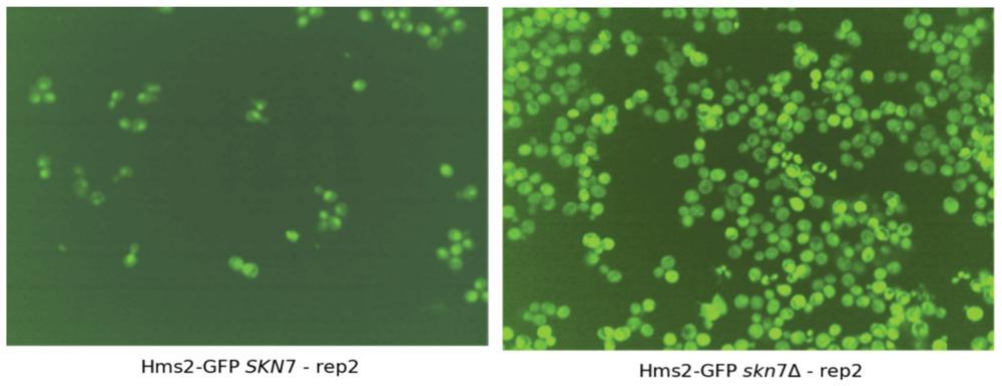
Micrographs of Hms2-GFP in the wild-type (*SKN7*) and deletion (*skn7*Δ) backgrounds. Micrographs of yeast cells in the wild-type background showed Hms2 localizing to the nucleus (depicted on the left) but changing localization to the cytoplasm upon the deletion of *SKN7* (depicted on the right). Hms2-Skn7 is an example of a pair of paralogous proteins that exhibited relocalization dependency.

The redistribution analysis revealed compensation and dependency mechanisms. Compensatory mechanisms involve the increase of protein abundance and/or the relocalization to a subcellular compartment of its paralog, while dependency mechanisms lead to the decrease in relative protein abundance and/or a subcellular localization different from its own and that of its paralog. An example of dependent redistribution is seen with the paralog pair, *SKN7-HMS2* (Dandage *et al*. 2025). Skn7 is a transcription factor needed for heat shock response to oxidative stress and osmoregulation (Raitt *et al*. 2000; Janiak-Spens *et al*. 2005), while Hms2 is similar to a heat shock transcription factor (Engel *et al*. 2022). Hms2 exhibits a localization change from the nucleus to the cytoplasm upon the deletion of *SKN7*, a pattern distinct from its endogenous localization and that of its paralog (Fig. 4).

### Integrating other studies into PARPAL

Genetic interactions, PPIs, and the localization of their interactors were predictive of redistribution. The PARPAL database thus includes data from other resources and studies to enhance the understanding of paralog functional divergence and redundancy, that are available in the “External data and resources” tab. Each paralog is hyperlinked to the SGD (Wong *et al*. 2023) which provides curated and detailed information on the gene sequence and function. The database also provides a link to the digenic and trigenic interaction analysis for each paralog pair (Kuzmin *et al*. 2020). Low trigenic interaction fraction characterizes divergent paralogs that exhibit more paralog-specific digenic interactions than trigenic interaction, and, for example, was observed for *SKN7-HMS2* (Kuzmin *et al*. 2020). Hms2 redistributed in response to *SKN7* deletion with a dependent relocalization from the nucleus to the cytoplasm, but Skn7 did not redistribute in response to *HMS2* deletion (Dandage *et al*. 2025), consistent with their functional divergence. A high trigenic interaction fraction characterizes functionally redundant paralogs that exhibit more trigenic interactions than digenic interactions and was observed for *GGA1-GGA2* (Kuzmin *et al*. 2020), which encode proteins that are involved in Golgi trafficking (Zhdankina *et al*. 2001). These paralogous proteins redistributed in response to each other's deletion, with Gga1 showing a compensatory relocalization from cytoplasm to Golgi in response to *GGA2* deletion and Gga2 showing a dependent protein abundance change in response to *GGA1* deletion (Dandage *et al*. 2025). Paralogs with a sparse number of digenic and trigenic interactions do not have a corresponding trigenic interaction fraction and are reported as “unclassified” in PARPAL and include *UPA1-UPA2* (Kuzmin *et al*. 2020), which encode methyltransferases involved in ribosome biogenesis (Ismail *et al*. 2022). These paralogous proteins redistributed in response to each other's deletion by relocalizing from the nucleus to the cytoplasm (Dandage *et al*. 2025). This functional information is unavailable when examining their digenic and trigenic interactions only. Another study provided a comprehensive dataset of PPI changes of proteins in response to their paralog deletion (Diss *et al*. 2017). This study defines compensation as when a protein expands its PPI network by gaining interactions of its paralog when that paralog is deleted and dependency as when a protein loses its PPI in response to the deletion of its paralog. One of the examined paralog pairs is *GSY1-GSY2* encoding glycogen synthases (Unnikrishnan *et al*. 2003). Despite not meeting the significance threshold for the redistribution score, which reflects the sensitivity limitation of the redistribution scoring approach, Gsy1 showed a significant protein abundance change, compensating for the loss of *GSY2* (Dandage *et al*. 2025). Gsy1 also showed a compensatory PPI change through an increase in its homodimer levels upon *GSY2* deletion (Diss *et al*. 2017). Gsy2 was classified as a redistributed protein, which compensates for the loss of *GSY1* by increasing its protein abundance (Dandage *et al*. 2025), although Gsy2 showed a dependent PPI with Glg1 upon *GSY1* deletion (Diss *et al*. 2017). Connecting the redistribution response to the changes in PPI offers mechanistic insight into the functional relationship between paralogous proteins. The third study explored responsiveness of paralogous proteins when one of the members of the pair is deleted in 2 types of growth conditions: rich and minimal media (DeLuna *et al*. 2010). Cue4 redistributed in response to *CUE1* deletion by increasing in protein abundance and changing in its subcellular localization from ER to the cytoplasm (Dandage *et al*. 2025). Consistent with our findings, Cue4 was upregulated upon *CUE1* deletion in rich growth media (DeLuna *et al*. 2010). The integration of different datasets provides insight into paralog compensation and dependency.

### Conclusion

We established a comprehensive dataset of protein dynamics of paralogs in the budding yeast *S. cerevisiae*, which captures how proteins respond to the deletion of their paralog in terms of their subcellular localization and abundance (Dandage *et al*. 2025). PARPAL provides a platform to access data and visualize ∼3,500 micrographs on the redistribution of 82 paralog pairs for a total of 328 query strains and ∼460,000 cells screened. PARPAL also provides additional information on the different predictors of redistribution, like genetics and PPIs by integrating other studies. Together the PARPAL database is a rich resource to provide valuable insight for other studies and expand this knowledge to high-order eukaryotes.

## Data Availability

All yeast strains are available upon request. Tables S1 to S5 and Data S1 and Data S2 are available on the PARPAL website https://parpal.c3g-app.sd4h.ca/ and have been deposited as part of the companion manuscript Dandage et al., *iScience* 2025: Mendeley Data, V1, doi: 10.17632/zx9xw7w28f.1. Supplemental material available at G3 online.
